# Multisystemic Consequences of Brain-Derived Neurotrophic Factor (BDNF) Haploinsufficiency in the SD-BDNFtm1sage Rat Model

**DOI:** 10.3390/ijms27135881

**Published:** 2026-06-30

**Authors:** Lucyna Mrówczyńska, Włodzimierz Mrówczyński

**Affiliations:** 1 Department of Cell Biology, Faculty of Biology, Adam Mickiewicz University in Poznań, Uniwersytetu Poznańskiego 6, 61-614 Poznań, Poland; lumro@amu.edu.pl; 2 Department of Neurobiology, Poznan University of Physical Education, Królowej Jadwigi St. 27/39, 61-871 Poznań, Poland

**Keywords:** BDNF haploinsufficiency, SD-BDNFtm1sage rat model, affective dysregulation, multisystem metabolic pathology, neurobiological stress vulnerability

## Abstract

Brain-derived neurotrophic factor (BDNF) is one of the most pleiotropic signaling molecules in mammalian biology, regulating processes ranging from neuronal survival and synaptic plasticity to metabolic homeostasis. Under physiological conditions, BDNF expression is tightly regulated; however, it may be disrupted by a variety of adverse factors, including chronic psychological stress, sleep deprivation, oxidative stress, inflammation, aging, and metabolic imbalance. Prolonged exposure to any of these factors can chronically reduce BDNF levels, contributing to numerous disorders whose systemic consequences remain difficult to define conclusively. This uncertainty arises because the available evidence is drawn from heterogeneous sources including many species, wild-type and various gene-knockout models, and pharmacological studies of differing specificity—yielding findings that are often inconsistent and difficult to compare. Consequently, the full spectrum of multisystemic effects resulting from long-term partial BDNF deficiency remains incompletely characterized. The SD-BDNFtm1sage rat line, developed by SAGE/Envigo/Inotiv using zinc finger nuclease technology, was created to fill this gap. Sprague–Dawley rats with a heterozygous genotype retain one functional allele of the *Bdnf* gene, resulting in a partial, permanent reduction in BDNF expression that persists throughout life. This chronic and moderate BDNF deficiency allows the animal to survive but is insufficient to maintain normal homeostasis, disrupting many physiological systems and behavioral responses. This review summarizes findings from studies using the SD-BDNFtm1sage rat line and shows that its phenotypic spectrum—susceptibility to mental disorders, sleep disturbances, metabolic abnormalities, altered nociception, and impaired neuromuscular adaptation—closely reflects the multisystemic consequences of chronic BDNF deficiency. This broad relevance makes the model particularly useful for research with potential medical applications.

## 1. Introduction

Brain-derived neurotrophic factor (BDNF), first isolated from pig brain as a distinct neurotrophin by Barde et al. [1], is currently one of the most extensively studied protein molecules in the mammalian central nervous system [2] due to its importance in the development and maintenance of normal brain functions [3], as well as its role in the regulation of metabolic processes across the lifespan [4,5].

During development, BDNF has been recognized as a key molecular regulator of neurogenesis [6], promoting neuronal proliferation and differentiation [7]. Furthermore, BDNF contributes to neuronal development [8], playing a role in the development of the soma [9], axons [10], and dendritic spines [11], as well as in the formation of appropriate connections in the developing nervous system [12].

In adults, BDNF is widely distributed throughout the brain. Its levels are particularly high in regions essential for synaptic plasticity, learning, memory, and neuroendocrine regulation [13]. The prefrontal cortex and the striatum are among the richest sources of BDNF. These structures support higher-order cognitive functions [14], including working memory [15], cognitive control [16], emotion regulation [17], extinction learning [18,19], decision-making [20], and the acquisition of motor skills [21]. High concentrations of BDNF are also found in the hippocampus. This region plays a key role in memory formation and plasticity [22], spatial learning and memory [23], and adult neurogenesis associated with pattern recognition and memory flexibility [24]. The amygdala and other limbic structures contain substantial amounts of BDNF as well. They are involved in affective processing [25], including the formation, storage, and retrieval of fear-related memories [26,27], as well as the encoding and consolidation of experiences into long-term memory [28,29]. The cerebellum is another important source of BDNF. It contributes to motor coordination and balance [30,31] and supports adaptive motor learning [32]. Finally, BDNF is present in the hypothalamus. This region regulates metabolism and homeostasis [33], including adaptive control of food intake, energy expenditure, body weight, and neuroendocrine secretion [34,35].

In these structures, BDNF levels during adulthood are strongly regulated by metabolic and hormonal factors [36,37], physical activity [5], and diet [38], enabling neurons both to carry out adaptive processes such as dendritic-spine formation, long-term potentiation, and activity-dependent synaptic remodeling and to integrate internal and external signals that maintain homeostasis.

BDNF exerts its biological actions primarily through the high-affinity TrkB receptor and the low-affinity p75^NTR^ receptor [39,40]. Activation of these receptors triggers cascades of intracellular signals, including MAPK, PI3K/Akt, and PLC-γ pathways [41], which collectively regulate dendritic growth, synaptic maturation, and long-term neuronal connectivity in the brain [42].

BDNF-TrkB signaling links neuronal activity to synaptic enhancement and structural remodeling of the neural network. In this way, it serves as a fundamental biological mechanism for translating environmental input into permanent changes in neural network function, governing multiple aspects of an organism’s behavior including learning, memory, mood, motor coordination, and the long-term regulation of body homeostasis [43,44,45,46]. Therefore, BDNF is a pleiotropic signaling protein that exerts numerous effects on various tissues and organs [47].

Conversely, numerous stressors reduce BDNF availability and/or impair TrkB-dependent plasticity in various structures of the nervous system. These include chronic psychological stress combined with prolonged exposure to glucocorticoids [48,49,50,51], sleep deprivation [52,53,54,55], oxidative stress resulting from mitochondrial dysfunction during periods of high energy demand, the excitotoxic activity of glutamate, hypoxia, ischemia, and various environmental factors [56,57,58], neuroinflammation induced by sustained microglial activation and prolonged release of pro-inflammatory cytokines such as interleukin-1β, tumor necrosis factor-α, and interleukin-6 [59,60], as well as a diet rich in fats and simple carbohydrates [61].

The neurobiological effects of such disruptions in the BDNF/TrkB signaling pathway depend on the brain region in which they occur. Changes in neural circuits in the prefrontal cortex and hippocampus underlie memory impairments, emotional dysregulation, and executive dysfunction—features frequently observed in stress-related mental disorders such as chronic insomnia, depression, addictions, and schizophrenia [62,63,64]. In the hypothalamus, disruption of the BDNF/TrkB signaling axis manifests as excessive appetite, reduced energy expenditure, and severe obesity [65,66,67].

However, the pathological effects of BDNF deficiency have rarely been the subject of comprehensive studies, and the existing literature consists mainly of independent studies focusing on specific brain regions or functional areas. This fragmented approach is to some extent methodologically justified due to area-specific patterns of BDNF expression and the diversity of BDNF/TrkB signaling pathways, which make it difficult to assess the systemic effects of BDNF deficiency within a single experiment.

Therefore, genetically modified animals appear to be the most powerful tool for systematically analysing the links between BDNF deficiency, impairments in neural circuit function, and behavioral or metabolic effects under controlled experimental conditions. Rodent models with a heterozygous deletion of the *Bdnf* gene are particularly relevant in this regard, as they reflect a moderate but persistent reduction in BDNF availability, often characteristic of human stress-related and metabolic disorders, by lowering BDNF protein levels by approximately 50% [68,69]. Furthermore, Monteggia et al. [70] and Edelmann et al. [71] have suggested that animals with partial BDNF deficiency represent a powerful preclinical tool for elucidating how chronically reduced neurotrophin expression shapes the organization of neural circuits, synaptic physiology, and behavioral responses. In addition, the deletion of the *Bdnf* gene in these animals is constitutive, resulting in reduced BDNF levels from conception through adulthood. Consequently, the phenotype of adult heterozygous animals reflects the combined effects of BDNF-dependent developmental abnormalities and persistent BDNF deficiency in the mature brain. Given the key role of BDNF during development, chronic deficiency of this factor may induce permanent changes in neural circuit architecture, making it difficult, in such models, to distinguish developmental effects from those emerging in adulthood.

In this context, Sprague–Dawley rats that are heterozygous carriers of a *Bdnf* gene deletion (SD-BDNFtm1sage), originally bred by SAGE Labs and subsequently maintained by Envigo/ Inotiv, provide a particularly valuable platform for studying the effects of reduced BDNF levels across multiple physiological systems and the susceptibility of these animals to disease. Their availability on the market with a clearly identified supplier and documented genetic lineage represents a significant methodological advantage, substantially reducing interpretive inconsistencies that have complicated studies of various gene-knockout models (ambiguous genotype reporting, incomplete documentation of parental strains, and interlaboratory variability in breeding and ablation procedures) [70,72]. A standardized genetic background also enables reliable interlaboratory comparisons and promotes the reproducibility of results across different independent research groups. However, despite its well-characterized genetic background, this strain remains relatively underreported in the literature, and the full spectrum of phenotypic effects resulting from the loss of a single *Bdnf* allele has not yet been systematically described in these animals. For these reasons, this review aims to summarize the results of previous studies conducted using the SD-BDNFtm1sage model, as well as to assess the biological and translational significance of the phenotype resulting from chronic BDNF deficiency.

Although the original studies sometimes describe these animals as “knockout rats,” they are in fact heterozygous and haploinsufficient: they retain one functional copy of the *Bdnf* gene and are therefore not complete (homozygous) knockouts, which are not viable. For precision, this review refers to them as heterozygous or haploinsufficient rats and reserves the term “knockout” for the complete, homozygous loss of BDNF.

## 2. Overview of the BDNF-Deficient Rat Model

At the molecular level, the rat *Bdnf* gene has a structure as complex as that of the human gene: numerous 5′ alternative non-coding exons, each with its own promoter, are spliced together with a single 3′ coding exon, which encodes the full-length preproBDNF protein. This conserved multi-promoter structure, combined with a high degree of coding sequence similarity, underscores the importance of BDNF research in rodents for translational studies. One significant difference between species is that the Val66Met polymorphism in humans (rs6265), which affects activity-dependent BDNF release and is associated with several neuropsychiatric traits, does not occur naturally in rats.

The SD-BDNFtm1Sage line was generated in a Sprague–Dawley rat genetic background by SAGE Labs using CompoZr zinc finger nuclease (ZFN) technology [70,72]. A pair of ZFNs targets the Bdnf protein-coding region, inducing a double-strand break that is repaired by non-homologous end joining (NHEJ). This results in a small deletion and a premature stop codon, leading to the production of a truncated, non-functional protein instead of mature BDNF. This procedure is sufficient to disrupt all BDNF production, because although the rat *Bdnf* gene contains multiple alternative 5′ non-coding exons driven by separate promoters, all transcripts share a common 3′ coding exon [73]. Consequently, a mutation in this shared exon affects every transcript variant, regardless of the promoter used, preventing functional BDNF expression from the mutated allele. As a result, homozygous rodent mutants are completely depleted of BDNF and die within the first few days after birth, whereas heterozygotes retain one intact allele and survive lifelong with an approximately 50% reduction in BDNF expression [see Envigo technical summary of the BDNF-KO rat model, which provides the most comprehensive overview of the homozygous and heterozygous phenotypes at: https://insights.envigo.com/hubfs/resources/data-sheets/envigo-gems-bdnf-ko-rat.pdf (accessed on 19 June 2026)].

### 2.1. Alterations in Organ Morphology and Body Composition

Gururajan et al. [74] reported reduced relative brain and pituitary weight in SD-BDNFtm1sage heterozygous rats compared to wild-type controls. Given BDNF’s role in the proliferation, differentiation, and survival of neurons in the cerebral cortex and hypothalamus (see Introduction), its chronic deficiency likely reduces brain mass relative to body weight. Moreover, the reduction in pituitary mass may reflect impaired trophic support for pituitary cells, with potential consequences for hypothalamic–pituitary–adrenal (HPA) axis function. Consistent with this interpretation, both St. Laurent et al. [75] and Grzelak et al. [76] reported significantly elevated body mass in heterozygous rats in the range of 15–30%, suggesting that metabolic dysregulation is a reproducible feature of this line. Grzelak et al. [77] further showed that liver weight was approximately 19% greater in heterozygous animals than in wild-type animals. Collectively, these findings indicate that BDNF haploinsufficiency alters body composition and organ morphology in ways that may affect systemic physiology. Nevertheless, heterozygous rats maintain a normal lifespan and do not display clearly visible behavioral abnormalities.

### 2.2. Tissue-Specific Alterations in BDNF and TrkB Expression

In heterozygous SD-BDNFtm1sage rats, mature BDNF was reduced by approximately 50% in the frontal cortex and dorsal hippocampus [78,79], and by 31% and 45% in the hippocampus and amygdala, respectively [80]. Serum BDNF was also markedly lower, with inter-study estimates ranging from 20% to 73% below wild-type levels [75,76], a variability likely reflecting differences in animal age and methodology.

Moreover, Grzelak et al. [76] also reported significant reductions in serum GDNF, NT-3, NT-4, and NGF (by 22%, 31%, 29%, and 37%, respectively), indicating broad downregulation of circulating neurotrophic support rather than an isolated BDNF deficit. These reduced serum levels of BDNF and other neurotrophic factors in heterozygous rats highlight the potential clinical utility of their peripheral measurement as a biomarker of central neurotrophic deficiency. This decrease in BDNF, GDNF, NT-3, NT-4, and NGF concentrations appears to be systemic in nature, as it was detected exclusively in serum, whereas concentrations in three hindlimb muscles (tibialis anterior, gastrocnemius, and soleus) remained unchanged [76]. The full panel of neurotrophins was not examined in any other tissue or in any other brain region in rats from the SD-BDNFtm1sage line. Because the mutation affects only BDNF, the reduced concentrations of other neurotrophic factors are likely secondary. They may arise from altered platelet content or release (platelets store most circulating BDNF and also transport other neurotrophins), from disrupted crosstalk between neurotrophic signaling pathways, or from systemic sequelae of BDNF deficiency, i.e., HPA-axis dysfunction, elevated glucocorticoids, and obesity, any of which could suppress circulating neurotrophic support.

Another issue concerns the cellular sources of circulating BDNF in these studies. Serum BDNF concentrations primarily reflect the large pool of this factor stored in the α-granules of platelets, whereas plasma contains only a small fraction of free BDNF derived from the brain, endothelium, and platelets [81]. Since platelets are the main reservoir of BDNF in the body [82,83], serum concentrations are strongly influenced by platelet count and platelet activation, while immune cells and the endothelium provide additional BDNF, particularly in inflammatory conditions [84]. However, no studies have been conducted in the SD-BDNFtm1sage rats’ line specifically regarding BDNF in platelets, peripheral blood mononuclear cells, or the vascular endothelium. The reported decreases in serum concentrations [75] were measured using a total serum immunoassay without distinguishing between these compartments. Determining whether the deficiency reflects altered platelet content, reduced production by immune or endothelial cells, or changes in release would clarify the biological significance of BDNF changes in peripheral blood in this model and remains an important direction for future research.

**Figure 1:**
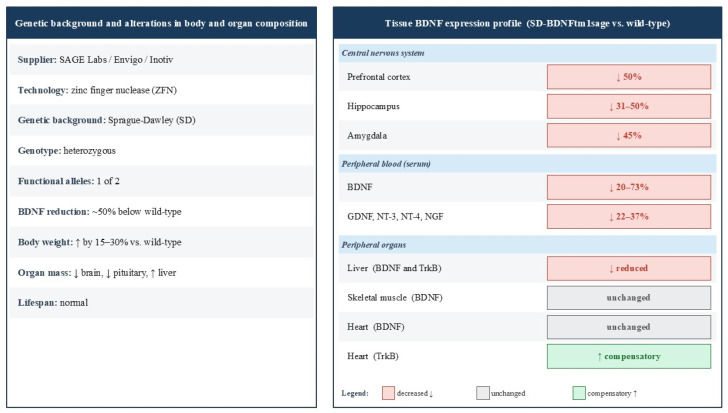
Characteristics of the SD-BDNFtm1sage heterozygous rat: genetic background and alternation in body and organ composition (**left panel**) and tissue-specific BDNF expression profile in compared to wild-type rat **(right panel**).

Hepatic BDNF and TrkB levels were also reduced [85], identifying the liver as a site of BDNF/TrkB-dependent metabolic regulation. In contrast, BDNF levels in hindlimb skeletal muscles (tibialis anterior, medial gastrocnemius, and soleus) were unchanged in all genotypes [75], suggesting that BDNF expression in skeletal muscles is regulated by contraction-driven autocrine or paracrine pathways rather than by the loss of a single allele of the *Bdnf* gene. Cardiac BDNF content was also unaffected while myocardial TrkB expression was elevated in heterozygous rats [85], consistent with compensatory receptor upregulation in response to chronically reduced ligand availability. Therefore, in heterozygous SD-BDNFtm1sage rats, a tissue-specific rather than uniform systemic reduction in BDNF expression was observed. This pattern reflects the variable dependence of individual organs on BDNF signaling and highlights that the physiological consequences of partial neurotrophin deficiency are not uniformly distributed across tissues (Figure 1). It should be noted that BDNF levels in this rat model have primarily been quantified as protein, while direct measurements of BDNF mRNA in this strain were reported by Sapio et al. [86], who performed RNA sequencing of dorsal root ganglia and the dorsal spinal cord in heterozygous and wild-type rats. However, no changes were found between heterozygous and wild-type rats, indicating that the haploinsufficiency phenotype reflects reduced functional protein rather than decreased transcript abundance.

## 3. Consequences of Imbalances in BDNF Processing and BDNF Deficiency

### 3.1. Cortico-Limbic System Dysfunction and Affective Dysregulation

Shirayama et al. [87] and Yang et al. [88] conducted studies on healthy Sprague–Dawley rats to understand how impairments in BDNF processing affect susceptibility to stress. Shirayama et al. [87] examined BDNF/proBDNF signaling in a learned helplessness model of depression characterized by motivational deficits and impaired escape behavior analogous to psychomotor retardation in clinical depression. Depression-like behavior was associated with region-specific imbalances: mature BDNF was reduced and proBDNF elevated in the medial prefrontal cortex (mPFC), CA3, and dentate gyrus of the hippocampus, while the opposite pattern was observed in the nucleus accumbens (NAc), indicating dysregulated BDNF processing within the mPFC–NAc circuit. Since proBDNF preferentially activates p75^NTR^, promoting synaptic weakening, the elevated proBDNF/BDNF ratio in the mPFC likely impaired active coping strategies. These changes were paralleled by reduced TrkB phosphorylation in the mPFC and hippocampus, and increased phosphorylation in the NAc. Bilateral infusion of the TrkB agonist 7,8-DHF restored TrkB phosphorylation and produced antidepressant-like effects, confirming the functional significance of these circuit-specific imbalances.

Yang et al. [88] extended this research by comparing preproBDNF, proBDNF, and BDNF pro-peptide expression across the mPFC, hippocampus, and NAc in stress-susceptible, stress-resilient, and unstressed rats. Susceptible animals showed markedly elevated levels of all three precursor forms in the mPFC, while resilient animals-maintained expression comparable to unstressed controls, suggesting that precursor accumulation in the mPFC is specifically linked to stress susceptibility. In the NAc, precursor levels were reduced only in susceptible rats, whereas hippocampal changes did not reliably distinguish between groups.

Importantly, in both studies, brain tissue from homozygous BDNF knockout rats served as a negative control to validate antibody specificity, confirming that full knockout abolishes all BDNF precursor forms.

Martis et al. [89] then demonstrated that this vulnerability can arise from genetic BDNF reduction alone, without any external stressor. SD-BDNFtm1sage rats exhibited a depression-like phenotype characterized by reduced sucrose preference (anhedonia) and heightened anxiety-like behaviors (reduced central zone activity in the open field test), while the forced swim test revealed no behavioral despair and cognitive functions remained intact. Molecularly, the prefrontal cortex showed upregulation of glucocorticoid receptor (GR), neuregulin 1 (Nrg1), and disrupted-in-schizophrenia 1 (Disc1), while hippocampal FK506 binding protein 5 (Fkbp5) was downregulated, with the prefrontal cortex exhibiting broader dysregulation than the hippocampus, identifying it as the primary site of molecular vulnerability.

Together, these three studies demonstrate that depression-like behavior and stress susceptibility reflect not simply a reduction in total BDNF availability, but a disruption of the regional balance between proBDNF and mature BDNF signaling. Stress susceptibility is characterized by precursor accumulation in the mPFC, shifting signaling toward p75^NTR^-mediated synaptic weakening and impairing active coping, while resilience depends on efficient proBDNF-to-mature BDNF conversion and sustained TrkB phosphorylation. BDNF genetic haploinsufficiency alone reproduces this same prefrontal vulnerability, establishing the prefrontal cortex as the primary convergence point of BDNF-dependent stress susceptibility with direct relevance to the pathophysiology of depression and treatment-resistant affective disorders.

Overall, these results indicate that susceptibility to mental disorders is determined not only by a decrease in total BDNF levels, but also by regional efficiency in the conversion of proBDNF to mature BDNF. In stress-susceptible animals, the accumulation of the precursor in the mPFC shifts signaling toward p75NTR-induced long-lasting depression, which impairs active stress coping independently of TrkB-regulated plasticity. This suggests that measuring total BDNF in serum or plasma may significantly underestimate the functional significance of altered neurotrophin processing, and that the proBDNF-to-mature BDNF ratio may serve as a more sensitive indicator of prefrontal circuit vulnerability than BDNF protein levels alone.

Gururajan et al. [74] examined the behavioral consequences of chronic corticosterone exposure in SD-BDNFtm1sage heterozygous and wild-type rats using the elevated plus maze, open field test, and forced swim test. Heterozygous rats showed a paradoxical anxiolytic-like response on the elevated plus maze and mildly reduced inner-zone activity in the open field, while no genotype or treatment effects emerged in the forced swim test, confirming an absence of behavioral despair. Hippocampal mature BDNF was reduced by ~50% in heterozygous rats, but corticosterone did not further suppress BDNF in either genotype, indicating that behavioral differences were attributable to the baseline genetic deficit rather than stress-driven BDNF suppression. The authors concluded that BDNF deficiency produces a context-dependent, test-specific pattern of stress susceptibility rather than a uniform affective phenotype.

Gururajan et al. [78] extended this investigation by assessing cognitive and sensorimotor functions using the Y-maze, novel object recognition, fear conditioning and extinction, and prepulse inhibition. Heterozygous rats showed impaired short-term spatial memory and reduced prepulse inhibition independent of corticosterone treatment, indicating that reduced BDNF availability disrupts spatial cognition and sensorimotor gating regardless of stress exposure. Chronic corticosterone impaired novel object recognition equally in both genotypes. The most significant finding was that fear extinction learning and recall were selectively disrupted by corticosterone in heterozygous rats only, identifying a gene-by-environment interaction with direct relevance to post-traumatic stress disorder (PTSD) and treatment-resistant depression.

Harris et al. [80] investigated the neural basis of these behavioral deficits using fMRI in awake, non-anesthetized rats. Wild-type animals showed robust activation of the left amygdala complex, insular cortex, and periaqueductal grey during conditioned fear expression, whereas heterozygous rats showed no significant activation of this circuitry. Peripheral BDNF (serum) was reduced alongside central levels (hippocampus, amygdala), with no compensatory upregulation of TrkB, and serum BDNF correlated with central measures, supporting its potential utility as a biomarker of central affective processing. These findings indicate that chronic BDNF deficiency disrupts coordinated amygdala-centered network dynamics underlying fear expression rather than attenuating activity within individual structures.

Overall, these studies show that BDNF haploinsufficiency produces a selective, context-dependent pattern of neurobiological vulnerability rather than a uniform affective phenotype. BDNF deficiency in the hippocampus has a genetic basis and is not related to stress; however, prolonged exposure to glucocorticoids selectively reveals a gene-environment interaction (impaired anxiety extinction) that directly contributes to the development of post-traumatic stress disorder (PTSD) and treatment-resistant depression.

Harris et al. [80] identified the neural basis of behavioral disorders, demonstrating that BDNF deficiency disrupts the coordinated dynamics of the network centered around the amygdala, rather than impairing the activity of individual structures. BDNF deficiency in the hippocampus likely underlies this neural network dysfunction by weakening the signal reaching the amygdala, which simultaneously impairs extinction learning and the coordinated activation of fear circuits. The correlation between serum BDNF levels and levels in the central nervous system further confirms that measurement of BDNF in peripheral blood is a clinically accessible biomarker of central affective circuit function.

St. Laurent et al. [75] investigated the role of BDNF in cocaine reward and relapse-like behavior using female heterozygous SD-BDNFtm1sage rats and wild-type controls. Animals were subjected to a conditioned place preference paradigm across four conditioning sessions with cocaine or saline, followed by eight days of extinction and a single priming injection to assess reinstatement of cocaine-seeking behavior. Serum BDNF levels confirmed lower concentrations in heterozygous rats prior to testing. Wild-type rats exhibited a clear preference for the cocaine-paired chamber on the first day of extinction, whereas heterozygous rats did not show a significant place preference. Following the reinstatement injection, wild-type rats resumed cocaine-seeking behavior, whereas no such reinstatement was observed in heterozygous rats, indicating that reduced BDNF expression impairs not only the acquisition of cocaine reward but also relapse-like responses following abstinence. Notably, only wild-type rats with high baseline serum BDNF showed significant place preference and reinstatement, suggesting that neuroplastic changes supporting cocaine reward require BDNF availability above a critical functional threshold.

This study demonstrated that reward processing deficits in SD-BDNFtm1sage rats are not limited to natural rewards such as sucrose [89] but extend to drug-related rewards, reflecting a generalized reduction in mesolimbic network plasticity. It also exposes a considerable paradox: while BDNF haploinsufficiency increases vulnerability to depression, anhedonia, and stress-related disorders [74,78,87], it also simultaneously confers protection against cocaine addiction suggesting that BDNF does not simply protect against all psychiatric disorders, but rather determines the responsiveness of specific brain circuits, which, depending on the situation, can be either beneficial or harmful. The correlation between baseline serum BDNF and addiction susceptibility further reinforces the biomarker utility of peripheral BDNF measurement established by Harris et al. [80].

### 3.2. Sleep Dysregulation

Garner et al. [79] investigated sleep–wake architecture and the homeostatic regulation of REM sleep in male and female heterozygous SD-BDNFtm1sage rats using cortical and hippocampal EEG following surgical electrode implantation. Post-recording tissue analysis confirmed ~50% reduction in BDNF protein in the frontal cortex alongside clear REM sleep disruption: fewer and shorter REM episodes, prolonged REM latency, reduced total REM duration, and more fragmented NREM sleep. Crucially, total NREM duration and slow-wave activity were preserved, indicating that NREM homeostatic mechanisms remain intact despite marked REM dysregulation. Under three hours of REM sleep deprivation, wild-type rats developed progressive REM pressure and a clear rebound, whereas heterozygous rats showed neither response, revealing a fundamental deficit in REM homeostatic regulation. No sex differences were observed in any sleep parameter.

REM sleep disturbances are a hallmark of depression, post-traumatic stress disorder (PTSD), and schizophrenia—disorders whose behavioral and molecular symptoms have been independently demonstrated in the same rat model [74,78,87]. Since REM sleep regulation depends on the same corticolimbic and hippocampal circuitry implicated in fear extinction, stress resilience, and reward processing, sleep dysregulation is best understood as a neurophysiological manifestation of the same underlying circuit-level deficit—likely forming a self-reinforcing cycle with prefrontal molecular dysregulation, glucocorticoid excess, and affective vulnerability rather than representing an independent finding.

It is worth noting that Garner et al. [79] reported no sex differences across all sleep parameters examined, including the number of REM sleep episodes, duration, latency to onset, and homeostatic rebound. This suggests that in the SD-BDNFtm1sage rat line, the functional impact of BDNF haploinsufficiency on sleep architecture is independent of sex, which distinguishes this model from other ones and justifies further research under conditions of chronic stress or hormonal stress.

### 3.3. Abnormalities in Epigenetic Programming

Paredes et al. [90] investigated the role of BDNF in the epigenetic programming of the hippocampus in newborns using two complementary models: SD-BDNFtm1sage rats and wild-type rats that received microinjections of tetrodotoxin (TTX) into the CA3 hippocampal region. Both treatments reduced BDNF levels in the hippocampus by ~45% and ~21%, respectively, and caused significant global DNA hypomethylation in the CA3 region during the first week after birth, as reflected by reduced 5-methylcytosine and 5-hydroxymethylcytosine immunoreactivity. These methylation deficits did not persist into adulthood, indicating that BDNF deficiency disrupts epigenetic programming within a specific early developmental period rather than causing permanent epigenetic changes. Despite the transient nature of these changes, both models demonstrated persistent behavioral abnormalities in adulthood characterized by impaired prepulse inhibition of the acoustic startle response—a well-known endophenotype of schizophrenia. The convergence of this phenotype in genetic and pharmacological models clearly indicated that BDNF-dependent epigenetic programming during the first week of life is a key mechanism linking early hippocampal dysfunction to behavioral pathology associated with schizophrenia in adulthood. Notably, this is the only study in the SD-BDNFtm1sage literature that directly addresses the developmental-versus-adult origin of the phenotype. By combining the constitutive genetic model with an acute postnatal TTX manipulation, and by demonstrating that the causal methylation changes are confined to the first postnatal week yet generate lifelong behavioural deficits, Paredes et al. [90] localise the critical BDNF-dependent insult to a discrete developmental window rather than to the mature circuit.

The findings of Paredes et al. [90] provide a significant reinterpretation of the adult phenotype described across studies of this rat line. Transient hippocampal DNA hypomethylation during the first postnatal week suggests that prefrontal transcriptional dysregulation (Disc1, Nrg1, GR) [85], impaired prepulse inhibition and fear extinction deficits [78], and amygdala network dysfunction [80] may reflect the lasting consequences of disrupted epigenetic programming during a critical developmental window rather than solely the chronic absence of BDNF in mature circuits. This interpretation does not undermine previous findings but reframes them: behavioral and molecular features attributed to adult BDNF deficiency may in part represent neurodevelopmental sequelae, particularly those manifesting under conditions of chronic stress. Consequently, the SD-BDNFtm1sage model may prove valuable not only for studying stress-related psychiatric disorders but also as a platform for neurodevelopmental research.

### 3.4. Dysfunction in Hepatic and Myocardial Metabolism

Grzelak et al. [77,85] demonstrated that BDNF haploinsufficiency impairs metabolic function in both the liver and heart. In the liver, heterozygous SD-BDNFtm1sage rats showed reduced expression of BDNF and TrkB, increased body and liver weight consistent with obesity and early-stage fatty liver disease, and impaired lipid metabolism—decreased total cholesterol and LDL alongside elevated triglycerides. Reduced ALAT and GGT activity indicated hepatocyte dysfunction resembling metabolic syndrome. Heart tissue showed a strikingly parallel profile: decreased cholesterol, LDL, ALAT, AST, and GGT, alongside decreased CK and CK-MB activity reflecting impaired ATP availability during myocardial contraction. Importantly, cardiac BDNF levels remained unchanged despite systemic depletion, whereas TrkB expression was elevated, suggesting a compensatory response. These results indicate that reduced BDNF signaling is associated with parallel metabolic and cardiovascular pathology in conjunction with the neurological dysfunctions described above—arguably the most unexpected systemic consequence of BDNF haploinsufficiency in this model.

The metabolic and cardiovascular pathology identified by Grzelak et al. [77,85] likely reflects the downstream systemic consequences of the same neurological deficit. Prefrontal molecular dysregulation [89] impairs HPA axis control, potentially driving peripheral metabolic disturbances, while glucocorticoid excess associated with heightened stress susceptibility [74] promotes hepatic lipid accumulation, dyslipidaemia, and cardiovascular dysfunction. REM sleep dysregulation [79] may further amplify these effects given the well-established links between sleep disruption and metabolic syndrome, collectively positioning hepatic and cardiac findings as systemic extensions of the neurological phenotype rather than isolated observations.

Five weeks of moderate-intensity endurance training produced only limited benefits. Hepatic BDNF, TrkB, and lipid profiles remained unchanged, although elevated ALAT, ASAT, and interleukin-6 (IL-6) levels suggested exercise-induced myokine signaling with partial metabolic activation. In the heart, only CK activity increased in both genotypes. These results indicate that exercise alone is insufficient to reverse the metabolic consequences of chronic BDNF deficiency.

Comparing the responses of the liver and heart to BDNF haploinsufficiency reveals a notable difference. In the liver, concentrations of both BDNF and TrkB protein were reduced [77], indicating parallel downregulation of both the ligand and the receptor, which prevents BDNF/TrkB signaling. In contrast, in the heart, BDNF levels remained unchanged, while TrkB expression was elevated [85], consistent with compensatory upregulation of the receptor in response to chronic reduction in BDNF availability in the circulatory system. This organ-specific discrepancy, the lack of compensation in the liver compared to receptor upregulation in the heart, suggests that the heart and liver differ fundamentally in their ability to mount an adaptive response to neurotrophin deficiency. However, the underlying mechanisms of these differences remain unknown, representing a gap in our understanding of the organ-specific consequences of systemic BDNF deficiency.

### 3.5. Neuromuscular Plasticity and Peripheral Neurotrophin Signaling

Grzelak et al. [76] examined the relationship between neurotrophin levels and spinal motoneuron excitability using intracellular recordings from lumbar motoneurons alongside neurotrophin measurements in serum and three hindlimb muscles (tibialis anterior, medial gastrocnemius, and soleus) in trained and untrained wild-type and heterozygous SD-BDNFtm1sage rats. Despite markedly reduced serum concentrations of BDNF, GDNF, NT-3, NGF, and NT-4 in heterozygous rats, motoneuron electrophysiological properties remained unchanged, indicating that circulating neurotrophin levels do not determine motoneuron excitability. Moderate-intensity endurance training did not restore serum BDNF levels in either genotype. However, it significantly increased BDNF and GDNF concentrations in fast-twitch muscles (tibialis anterior and medial gastrocnemius) across both genotypes. This pattern indicates that exercise-induced neurotrophin upregulation in skeletal muscle occurs independently of the BDNF genotype.

Training also reduced fast motoneuron excitability, reflected by decreased input resistance in both genotypes. These findings identify muscle-derived rather than circulating neurotrophins as the primary drivers of exercise-induced motoneuron plasticity, demonstrating that BDNF plays a significant functional role in neuromuscular adaptation beyond its well-established actions in the central nervous system.

While serum BDNF reflects central BDNF deficiency [80], it does not predict neuromuscular function. These findings are complementary rather than contradictory: serum BDNF is a valid biomarker of central circuit dysfunction, but its functional role is tissue specific. Moreover, exercise restores neuromuscular plasticity through local muscle-derived neurotrophin signaling—a mechanism that partially compensates for the genetic deficiency in the neuromuscular compartment but does not extend to the hepatic and cardiac tissue, where training effects remained limited [77,85]. These findings give physical exercise new significance as a therapeutic strategy in the context of genetic BDNF deficiency. Contrary to earlier predictions, endurance training does not act as a universal method for restoring neurotrophin levels [91]. Instead, it activates contraction-dependent pathways that stimulate neurotrophin synthesis in skeletal muscle. These mechanisms operate independently of BDNF genotype and remain fully effective even when a single *Bdnf* allele is lost.

The resulting benefits are limited to the neuromuscular compartment: the excitability of motoneurons is modulated, and local neurotrophin production is upregulated, but this locally induced response is insufficient to compensate for the central neurotrophin deficit, normalize serum BDNF levels, or reverse liver and heart pathology. This model thus reveals the limits of the effectiveness of physical exercise, which has direct implications for the design of therapeutic protocols: physical activity alone is unlikely to constitute an effective therapy in states of chronic BDNF deficiency and should be considered a supplement, not a substitute, for interventions targeting central neurotrophin signalling. However, it should be emphasized that this conclusion applies only to short-term, moderate-intensity endurance training programs. Programs that differ in intensity, duration, or age of initiation, as well as other methods (e.g., strength training, environmental enrichment, or physical exercise combined with pharmacological treatment), have not been studied in this context and may produce different results.

### 3.6. Multilevel Regulation of Nociceptive Sensitivity

Xia et al. [92] investigated the role of BDNF in cross-sensitization between the colon and bladder in wild-type and heterozygous SD-BDNFtm1sage rats. TNBS-induced colitis increased CREB phosphorylation in bladder afferent neurons via the TrkB–PLCγ–CaMKII–CREB cascade in wild-type rats, while heterozygous rats showed significantly attenuated neurophysiological and urodynamic responses—including reduced urinary frequency and increased voided volume—identifying endogenous BDNF as a key paracrine mediator of intraganglionic cross-organ sensitization.

Liu et al. [93] examined BDNF-mediated spinal cord sensitization during colitis, demonstrating that TNBS-induced colitis increased NR1 subunit phosphorylation in the dorsal horn—an effect attenuated both by BDNF-neutralizing antibody and in heterozygous rats. Signal transduction analysis identified converging PLCγ/PKC and PI3K/Akt pathways as responsible, with PI3K/Akt representing a novel parallel mechanism distinct from classical PKC-mediated NR1 phosphorylation.

Sapio et al. [86] demonstrated that BDNF haploinsufficiency elevates pain thresholds in both humans and rats. WAGR syndrome patients with heterozygous BDNF deletion rated heat and cold stimuli as significantly less painful, while heterozygous rats showed prolonged withdrawal latencies and reduced Aδ fiber reactivity. Transcriptomic analysis pointed to spinal rather than peripheral mechanisms, with glial and interferon signaling pathways implicated. Together, these studies delineate a coherent BDNF-dependent nociceptive pathway: peripheral DRG sensitization via TrkB–PLCγ–CaMKII–CREB [92], central dorsal horn sensitization via NMDA receptor phosphorylation [93], and system-level pain threshold modulation through spinal processing [86]—confirming BDNF as a critical multilevel regulator of nociceptive sensitivity. The TrkB–PLCγ cascade identified here operates within the same signaling framework implicated in central BDNF deficiency, suggesting that disrupted TrkB-mediated plasticity is a unifying mechanism across central and peripheral manifestations of BDNF haploinsufficiency. Furthermore, the finding that haploinsufficiency raises pain thresholds in both rats and humans broadens the biomarker utility of peripheral BDNF measurement beyond affective circuit function [79] to nociceptive processing.

Among all the findings described in studies of the SD-BDNFtm1sage model, the observation of elevated pain thresholds reported by Sapio et al. [86] holds a unique position in the context of clinical applications, as it is the only finding that has been directly replicated in humans. Patients with WAGR syndrome, who are carriers of a heterozygous *Bdnf* gene deletion, exhibited reduced pain perception in response to thermal and cold stimuli, reflecting the prolonged withdrawal times and impaired fiber reactivity observed in heterozygous rats. This cross-species convergence significantly enhances the value of the SD-BDNFtm1sage model as an exceptionally well-validated translational platform for research on BDNF deficiency.

## 4. Limitations and Methodological Considerations

Several limitations of the underlying evidence base and of the present review should be acknowledged. The literature on the SD-BDNFtm1sage line is small and methodologically heterogeneous: the available studies differ in sample size, sex inclusion, age at testing, housing and husbandry, stress exposure, behavioural protocols, the timing of tissue collection, and assay methodology. Such heterogeneity is particularly consequential for BDNF, whose measured levels are highly sensitive to pre-analytical and contextual factors including circadian timing, recent physical activity, acute and chronic stress, diet, platelet activation, and sample handling. The convergence of results highlighted in this review should therefore be viewed as a qualitative phenomenon intended to inform hypothesis generation.

It should be noted that several of the mechanisms discussed here were not confirmed in the heterozygous rat line used in this study. Experiments conducted by Shirayama et al. [87] and Yang et al. [88] were conducted on wild-type Sprague–Dawley rats, and homozygous tissue with the *Bdnf* gene knocked out served only as an antibody control. Their results were included to broaden the interpretive context, but they do not demonstrate the presence of these mechanisms in SD-BDNFtm1sage heterozygotes. Table 1 provides a detailed summary of the model, genotype, and intervention for each of the cited studies.

**Figure 2:**
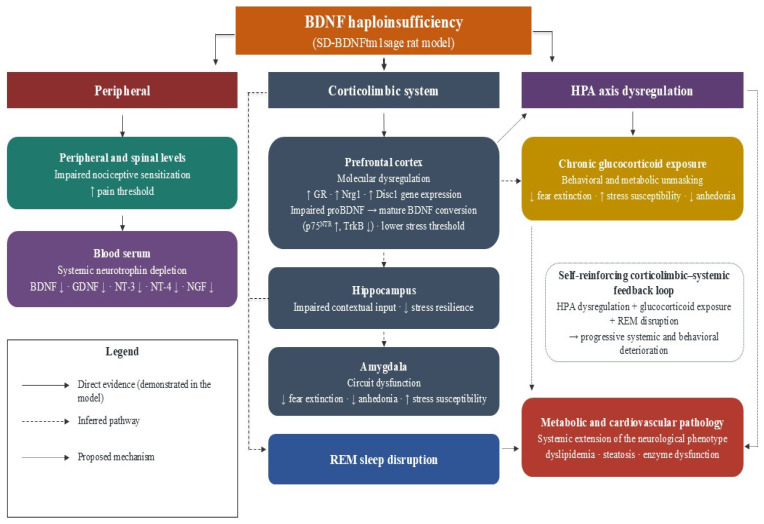
Self-reinforcing corticolimbic–systemic feedback loop in SD-BDNFtm1sage rats. Prefrontal BDNF deficiency is proposed to drive HPA-axis dysregulation, glucocorticoid excess, and REM-sleep disruption, which may in turn exacerbate peripheral metabolic and cardiovascular pathology. The diagram reflects an integrative hypothesis rather than a proven causal pathway.

The feedback loop between the cortical-limbic and the systemic systems, proposed in Section 3 and illustrated in Figure 2, should be regarded as an integrative hypothesis rather than a proven causal pathway. Establishing causality will require specifically designed experiments, including long-time studies, tissue-specific or time-controlled rescue of TrkB modulation, endocrine profiling, and formal analyses.

It should also be emphasized that the certainty of translational inferences is not uniform across all studies included in this review. It is strongest in the case of nociception, where reduced pain sensitivity has been consistently demonstrated in heterozygous rats and in humans with WAGR deletions who express BDNF [86]. It is moderate for affective, sleep, and reward phenotypes, which are well characterized in rats but rely on analogies to corresponding states in humans. In contrast, it is weakest for findings regarding metabolism and the cardiovascular system, which come from a small number of studies that have not been independently verified in relation to human diseases.

Finally, the phenotypes described in this review may have multiple underlying explanations. Because the deletion is constitutive, some behavioral and metabolic features in adult individuals may result from developmental programming or compensatory mechanisms rather than from a persistent BDNF deficiency in mature tissue (see Introduction and Section 3.3). Furthermore, increased body weight, altered body composition, fatty liver, reduced brain and pituitary mass, and possible neuroendocrine differences in this strain may confound behavioral measures dependent on locomotion, motivation, or body weight, although strain-specific effects cannot be ruled out. Therefore, considerable caution should be exercised before attributing a given behavioral observation or metabolic outcome solely to BDNF signaling.

It should also be emphasized that although the SD-BDNFtm1 line is derived from the Sprague–Dawley strain with a low degree of inbreeding, it nevertheless exhibits some genetic heterogeneity, and the deliberate mutation does not rule out the possibility of unknown changes introduced during genome editing. Therefore, as with all genetically modified animals, this model also raises ethical concerns that require strict adherence to the 3Rs principle (replacement, reduction, refinement) and careful monitoring of animal welfare.

Additionally, a reduction of approximately 50% in this highly pleiotropic neurotrophin does not allow for a full understanding of the biology of BDNF and its role in development, cell-type-specific signaling, the proBDNF/mBDNF balance, or gene-environment interactions. For these reasons, the SD-BDNFtm1 line should therefore be viewed as a useful starting point for analysing BDNF-dependent mechanisms, rather than as a comprehensive substitute for human disease.

The heterozygous rat with BDNF deficiency described in this review shares several key features with mouse models of BDNF haploinsufficiency. In both groups, an approximately 50 percent decrease in BDNF levels is observed, along with impairments in cognitive and affective functions [68,69,74,89]. However, there are two significant differences that limit the possibility of direct interspecies comparisons. First, mice exhibit marked hyperactivity [66], whereas rats are characterized by reduced motivation to explore [78]. Second, in mice, BDNF haploinsufficiency causes severe, late-onset obesity due to hyperphagia [65,94], which can be reversed by BDNF administration [66]. Despite the analogous genetic alteration, this obesity phenotype has not been described in heterozygous rats. These discrepancies indicate that the behavioral and metabolic consequences of partial BDNF loss manifest differently in these two species. Therefore, caution should be exercised when generalizing the results of studies conducted in different models of BDNF deficient rodents.

## 5. Conclusions

Studies conducted on the SD-BDNFtm1sage heterozygous rat model have shown that a ~50% reduction in BDNF expression is sufficient to induce a multisystemic phenotype that exceeds expectations from a single-allele deletion. The resulting deficits involve the central nervous system (affective dysregulation, cognitive impairments, REM sleep disturbances, and abnormalities in epigenetic programming), the peripheral and spinal nervous systems (nociception and cross-sensitization of visceral, spinal signaling), the neuromuscular system (motor neuron excitability), and peripheral organs (liver, heart) (Table 1). Importantly, BDNF reduction involves many tissues: significant deficits are observed in serum, prefrontal cortex, hippocampus, amygdala, and liver; whereas skeletal muscle and cardiac BDNF levels remain unchanged. This pattern indicates differential organ vulnerability rather than global neurotrophin depletion, supporting the model’s value for studying partial and chronic BDNF deficiency.

The prefrontal cortex is the primary site of molecular dysregulation, marked by increased expression of GR, Nrg1, and Disc1. These changes do not produce a uniform psychiatric profile but lower the threshold for stress sensitivity, so that only chronic glucocorticoid exposure unmasks impaired anxiety extinction, stress susceptibility, anhedonia, and disrupted REM sleep. In parallel, hippocampal BDNF deficiency and amygdala dysfunction disrupt the network dynamics underlying fear expression, extinction learning, and resilience. These effects are not explained by reduced BDNF availability alone: Shirayama et al. [87] and Yang et al. [88] show that stress susceptibility also depends on regional proBDNF maturation, since impaired conversion of proBDNF to mature BDNF in the medial prefrontal cortex shifts signaling toward p75NTR-mediated synaptic weakening. BDNF deficiency is therefore best understood as an imbalance between synaptic strengthening and weakening, with impaired TrkB signaling as the central pathway linking central and peripheral symptoms.

Beyond the nervous system, concurrent hepatic and myocardial changes confirm that BDNF signaling is integral to systemic metabolic homeostasis. These peripheral effects are not isolated: HPA-axis dysregulation, glucocorticoid excess, and REM sleep disturbance likely interact to aggravate metabolic and cardiovascular pathology, making liver and cardiac dysfunction systemic manifestations of the neurological phenotype. In the sensory domain, BDNF deficiency attenuates nociceptive sensitization at both peripheral and spinal levels, consistently raising pain thresholds in rat and human models and identifying BDNF as a multilevel regulator of nociception.

Two findings have practical relevance. First, serum BDNF reliably reflects central deficiency and correlates with affective-circuit function and nociceptive sensitivity; together with the parallel downregulation of GDNF, NT-3, NT-4, and NGF, this points to a systemic neurotrophin deficit and supports peripheral BDNF as a candidate biomarker of broader neuropsychiatric, metabolic, and pain-related dysfunction. This potential must, however, be read cautiously. The data establish biological validity and—in rats—analytical detectability, but not clinical utility: serum BDNF is strongly affected by platelet count, physical activity, circadian rhythm, and sampling method, and rat-tissue correlations do not confirm human relevance. Analytical accuracy, biological validity, and clinical utility must therefore be established independently before any clinical use.

Second, moderate-intensity endurance training selectively restores neuromuscular plasticity through a local pathway involving muscle-derived neurotrophins, without normalizing serum BDNF, central circuit integrity, or hepatic and cardiac homeostasis. Exercise alone is thus insufficient, and genetic BDNF deficiency likely requires a combined strategy targeting central neurotrophin signaling, neuroendocrine dysregulation, and peripheral metabolism simultaneously.

Epigenetic data suggests a developmental reinterpretation of the adult phenotype. Transient DNA hypomethylation in the hippocampus of newborn SD-BDNFtm1sage rats indicates that the adult behavioral and molecular profile reflects not only chronic deficiency in mature circuits but also the lasting consequences of disrupted early epigenetic programming. The convergence of identical behavioral abnormalities in genetic and pharmacological models confirms that BDNF-dependent epigenetic programming in newborns links early hippocampal dysfunction to adult psychiatric pathology, broadening the model’s relevance for neurodevelopmental research.

The model also exposes a functional paradox: BDNF haploinsufficiency increases susceptibility to depression, post-traumatic stress disorder (PTSD), and stress-related phenotypes while reducing cocaine reward, relapse propensity, and nociceptive sensitivity. BDNF thus acts as a circuit- and context-dependent regulator of neurobiological sensitivity rather than a uniform resilience factor. Clinically, indiscriminate restoration of BDNF signaling could yield divergent outcomes across affective, reward, and sensory systems, underscoring the need for network-specific therapeutic strategies.

Taken together, the genetically well-characterized and commercially available heterozygous SD-BDNFtm1sage rat is a robust translational tool for studying chronic partial BDNF deficiency, with a phenotypic spectrum that mirrors the multisystemic consequences seen in human disease. Its phenotypic complexity is not a limitation but its principal strength, revealing BDNF as a pleiotropic regulator of systemic homeostasis—from epigenetic programming and synaptic plasticity to organ metabolism and sensory processing. 

## Figures and Tables

**Table 1:** Spectrum of behavioral, physiological, and metabolic phenotypes associated with BDNF haploinsufficiency in the SD-BDNFtm1sage heterozygous rat model.

Source	Primary Model	Study Design	Primary Endpoint	Principal Finding	Translational Relevance
**Cortico-limbic system dysfunction and affective dysregulation**					
Shirayama et al. (2015) [87]	Wild-type Sprague–Dawley (primary model) Homozygous BDNF-KO is used only as antibody control	Observational with pharmacological rescue Learned-helplessness depression model TrkB-agonist intervention	BDNF/proBDNF and TrkB phosphorylation mPFC, hippocampus (CA3, DG), nucleus accumbens (NAc)	↓ mature BDNF and TrkB phosphorylation in mPFC, CA3, DG ↑ proBDNF in mPFC ↑ mature BDNF in NAc	TrkB agonist 7,8-DHF restored TrkB phosphorylation and produced antidepressant effects Supports BDNF–TrkB as an antidepressant target
Yang et al. (2016) [88]	Wild-type Sprague–Dawley (primary model) Homozygous BDNF-KO is used only as antibody control	Observational, no intervention Learned-helplessness model Stress-susceptible vs resilient comparison	preproBDNF/proBDNF/BDNF pro-peptide and proBDNF→BDNF conversion mPFC, hippocampus (CA1, CA3, DG), NAc	↑ preproBDNF, proBDNF, pro-peptide in mPFC of susceptible rats ↓ proBDNF in NAc Impaired mPFC proBDNF→BDNF conversion	Region-specific proBDNF processing distinguishes stress susceptibility Candidate vulnerability marker for depression
Martis et al. (2019) [89]	SD-BDNFtm1sage primary model	Observational, no intervention	PFC and hippocampus Affective behavior (anhedonia, anxiety) and stress-gene expression	Anhedonia (↓ sucrose preference) and anxiety-like behavior ↑ GR, Nrg1, Disc1 in PFC ↓ Fkbp5 in hippocampus	Links BDNF haploinsufficiency to schizophrenia- and stress-related gene pathways
Gururajan et al. (2014) [74]	SD-BDNFtm1sage primary model Wild-type Sprague–Dawley controls	Observational with chronic corticosterone challenge Genotype × stress design	Hippocampal BDNF Anxiety- and depression-related behavior (EPM, open field, FST)	~50% ↓ hippocampal BDNF Paradoxical anxiolytic effect on elevated plus maze Mild open-field anxiety; no forced-swim effect	Corticosterone did not further lower BDNF; behavioral effects context-dependent
Gururajan et al. (2015) [78]	SD-BDNFtm1sage primary model Wild-type Sprague–Dawley controls	Observational with chronic corticosterone challenge Genotype × stress design	Cognition (Y-maze, novel-object recognition), sensorimotor gating (PPI), fear extinction	Impaired spatial memory ↓ prepulse inhibition Fear-extinction deficit emerging only under corticosterone	Corticosterone selectively impaired fear extinction in heterozygous rats Gene × stress model of stress-related disorders
Harris et al. (2016) [80]	SD-BDNFtm1sage primary model Wild-type Sprague–Dawley controls	Observational, no intervention Functional MRI of fear circuitry	activity in amygdala, granular insular, periaqueductal gray during conditioned fear Tissue and serum BDNF	↓ BDNF (hippocampus 31%, amygdala 45%, serum 73%) Abolished fear-circuit activation	Serum BDNF as an accessible peripheral marker of central fear-circuit dysfunction
St. Laurent et al. (2013) [75]	SD-BDNFtm1sage primary model Wild-type Sprague–Dawley controls	Observational, no intervention Reward/addiction behavioral paradigm	Reward circuitry (VTA, NAc, PFC) Serum BDNF; cocaine-reward behavior	↓ serum BDNF Impaired cocaine-reward acquisition; abolished cocaine-seeking reinstatement Impaired natural and drug reward processing	BDNF haploinsufficiency dampens reward and relapse behavior Relevance to addiction vulnerability
Garner et al. (2018) [79]	SD-BDNFtm1sage primary model Wild-type Sprague–Dawley controls	Observational with REM-deprivation challenge EEG/EMG sleep recording	Sleep–wake architecture (EEG/EMG) REM/NREM dynamics and homeostatic REM rebound	~50% ↓ BDNF Fewer and shorter REM episodes; prolonged REM latency Fragmented NREM; abolished homeostatic REM rebound	BDNF gene dosage shapes REM homeostasis Links to sleep disturbance in mood disorders
**Abnormalities in epigenetic programming**					
Paredes et al. (2021) [90]	SD-BDNFtm1sage primary model Wild-type Sprague–Dawley (TTX comparison)	Developmental/observational Neonatal epigenetic profiling with adult behavioral follow-up	Neonatal hippocampal CA3 DNA methylation Adult prepulse inhibition	~45% ↓ hippocampal BDNF Global DNA hypomethylation in CA3 during first postnatal week Aberrant prepulse inhibition in adulthood	Early BDNF deficiency reprograms the epigenome with lifelong behavioral consequences
**Dysfunction in hepatic and myocardial metabolism**					
Grzelak et al. (2023) [77]	SD-BDNFtm1sage primary model Wild-type Sprague–Dawley controls	Observational with endurance-training intervention Genotype × exercise design	Hepatic metabolism, lipids, liver enzymes and BDNF/TrkB signaling	↓ hepatic BDNF and TrkB ↑ body and liver weight; hepatic steatosis ↓ CHOL/LDL, ↑ TG; ↓ ALAT and GGT	Training raised ALAT/ASAT and IL-6 in heterozygous rats only; BDNF/TrkB and lipids unchanged Limited exercise rescue of the hepatic phenotype
Grzelak et al. (2024) [85]	SD-BDNFtm1sage primary model Wild-type Sprague–Dawley controls	Observational with endurance-training intervention Genotype × exercise design	Myocardial metabolism, cardiac enzymes/lipids and BDNF/TrkB signaling	Cardiac BDNF unchanged but ↑ TrkB (compensatory) ↓ myocardial CHOL, LDL, ALAT, ASAT, GGT, CK, CK-MB	Training raised CK in both genotypes only Tissue-specific cardiac adaptation to BDNF deficiency
**Neuromuscular plasticity and peripheral neurotrophin signaling**					
Grzelak et al. (2023) [76]	SD-BDNFtm1sage primary model Wild-type Sprague–Dawley controls	Observational with endurance-training intervention Electrophysiology and neurotrophin assays	Lumbar motoneuron electrophysiology Hindlimb muscle and serum neurotrophins	Motoneuron electrophysiological properties unchanged Normal muscle neurotrophin levels ↓ serum neurotrophins	Training ↑ BDNF/GDNF in fast muscles and ↓ fast-motoneuron excitability regardless of genotype Exercise-driven peripheral plasticity is maintained
**Multilevel regulation of nociceptive sensitivity**					
Xia et al. (2016) [92]	SD-BDNFtm1sage primary model Wild-type Sprague–Dawley controls	Interventional disease model (TNBS colitis) Colon-to-bladder cross-sensitization paradigm	Dorsal root ganglion signaling Bladder afferent (cross-organ) function	Attenuated colitis-induced CREB phosphorylation in bladder afferents ↓ bladder overactivity Impaired TrkB–PLCγ–CaMKII–CREB signaling	Partial BDNF loss is sufficient to blunt cross-organ visceral sensitization Target for visceral pain/overactive bladder
Liu et al. (2015) [93]	SD-BDNFtm1sage primary model Wild-type Sprague–Dawley controls	Interventional disease model (TNBS colitis) Spinal sensitization with antibody comparison	Spinal dorsal-horn NMDA-receptor (NR1) signaling (L1–S1)	Attenuated colitis-induced NR1 Ser^896^ phosphorylation Impaired PLCγ/PKC and PI3K/Akt cascades	BDNF-neutralizing antibody and heterozygous line both blunted spinal sensitization Supports BDNF as a central-sensitization target
Sapio et al. (2019) [86]	SD-BDNFtm1sage primary model plus WAGR-syndrome human subjects Wild-type Sprague–Dawley rats and healthy humans as controls	Cross-species observational heterozygous rat vs WT and human WAGR (BDNF-haploinsufficient) vs healthy	DRG/spinal nociceptor function Thermal and mechanical pain thresholds; spinal transcriptome	↑ heat/cold withdrawal and von Frey thresholds ↓ Aδ and C-fiber responsiveness Glial/interferon transcriptomic shifts; DRG nociceptor populations intact	Haploinsufficiency raises pain threshold without abolishing nociception Rat findings mirror human WAGR— a direct translational bridge

Abbreviations: SD-BDNFtm1sage heterozygous rats used as the primary experimental model (green cell). Wild-type Sprague–Dawley rats used as the primary experimental model (blue cell). TTX—tetrodotoxin; FST—forced-swim test; EPM—elevated plus maze; PPI—prepulse inhibition; DRG—dorsal root ganglion; VTA—ventral tegmental area; NAc—nucleus accumbens; mPFC/PFC—medial prefrontal cortex; CA1/CA3—hippocampal subfields; DG—dentate gyrus; GR—glucocorticoid receptor; Nrg1—neuregulin 1; Disc1—disrupted-in-schizophrenia 1; Fkbp5—FK506-binding protein 5; CHOL—cholesterol; LDL—low-density lipoprotein; TG—triglycerides; ALAT/ASAT—alanine/aspartate aminotransferase; GGT—gamma-glutamyl transferase; CK/CK-MB—creatine kinase (MB isoform); IL-6—interleukin 6; 7,8-DHF—7,8-dihydroxyflavone; arrows pointing down (↓)—decrease; arrows pointing up (↑)—increase.

## Data Availability

No new data were created or analyzed in this study. Data sharing is not applicable to this article.
